# Analysis of food sources and nutrient intakes of selected breastfeeding mothers in Metro Manila, Philippines

**DOI:** 10.1186/s40795-022-00502-1

**Published:** 2022-01-18

**Authors:** Imelda Angeles-Agdeppa, Ma. Rosel S. Custodio, Keith V. Tanda

**Affiliations:** Food and Nutrition Research Institute, Department of Science and Technology, Taguig, Philippines

**Keywords:** Breastfeeding, Maternal Nutrition, Food Source, Nutrient Intake

## Abstract

**Background:**

This study evaluated the food and nutrient intakes of selected breastfeeding mothers and identified the top food sources of nutrient intakes.

**Methods:**

This is a cross-sectional, non-interventional study conducted in one of the private medical centers in Metro Manila, Philippines. Participants: The sample size included 70 mothers of healthy, term, exclusively breastfed infants aged 21–26 days at enrollment. Mothers were scheduled to visit the clinic at days 1, 22, 57, and day 90.Similarly, food diaries were obtained during these periods with a 3-day food record per clinic visit totaling to 12 records per mother at the end of 90 days. At every clinic visit, the records were validated face – to – face by the registered nutritionist – dietitians. An extension of the mother’s participation until day 155 was implemented for the collection of clinical outcomes used by Pediatricians. Mean food intakes were calculated. A PC-Software for Intake Distribution Estimation (PC-SIDE) program was used in the estimation of inadequate intakes. Socio-economic status was collected using standard questionnaires. Weight and height were measured using standard techniques to compute for BMI.

**Results:**

Anthropometric results for the breastfeeding mothers reported a mean weight of 56.9 kg (SE = 1.3) and a mean height of 152.4 cm (SE = 0.6). In terms of body mass index (BMI), 8.6% were chronic energy deficient, and 34.3% were overweight while 12.9% were obese. Mean energy intake of breastfeeding mothers was 2516.7 kcal/day, which was 28.6% higher than the EER of 1957 kcal/day. Mean protein intake was 78.4 g/day, which was 37% inadequate while fat intake as percentage of total energy was excessive by 4%. Nutrient inadequacy is high for almost all nutrients: iron (99%), folate (96%), riboflavin (39%), vitamin B6 (63%), vitamin B12 (46%) and thiamine (22%). The top 5-food sources with highest percent contribution to energy are rice (43.1%), bread (8.1%), pork (7.7%), powdered milk (5.9%), and sweet bakery products (5%).

**Conclusions:**

There is a high prevalence of protein and micronutrient inadequacies in the diet of breastfeeding mothers. The prevalence of overweight and obesity is high among breastfeeding mothers. These findings might be explained by the low variety and nutrient-poor foods consumed by the breastfeeding mothers. Understanding the major food sources of nutrient intake of breastfeeding mothers could be used to intensify if not craft interventions to address the nutrient gaps. Improving the maternal nutrition may contribute to having quality breastmilk to infants.

**Supplementary Information:**

The online version contains supplementary material available at 10.1186/s40795-022-00502-1.

## Background

The nutritional needs of breastfeeding mothers are increased during this stage of lactation to support the high nutritional needs of both the mother and the child. The energy requirement is increased by 450 to 500 kcal/day [1]. As a result of increased maternal nutrient needs due to milk production, inadequacy of the maternal dietary intake may result in the mobilization of nutrient stores resulting in loss of essential nutrients. Consistently not meeting recommended energy and nutrient needs will lead to either undernutrition and at times when the mothers’ consumption behaviors are directed towards energy-dense foods, this can also lead to overnutrition and increased risk for metabolic diseases. However, changes in body weight and composition among breastfeeding mothers within a diverse population are highly variable [2]. Several studies have supported that the nutrient intakes of mothers affect the quality of the breastmilk that is passed down to the infant [3]. In previous literatures, it has been shown that some metabolic pathways enable human milk components through maternal dietary intakes particularly the fatty acids and fat, water-soluble vitamins, and other vitamins such as vitamins A, C, B-6, and B-12 [2] [4]. Moreover, the mineral content of human milk is generally regarded as less compared to maternal dietary intakes [3].

In the Philippines, a survey by the Food and Nutrition Research Institute determined that about 12.5% of breastfeeding mothers have been reported to be chronically energy deficient, whereas 21.8% are overweight and obese [5]. In addition, the incidence of anemia was higher among adult mothers 19 years old and above who were breastfeeding (17.3%) relative to their younger counterparts aged 18 years old and below (9.8%) [5]. In the same survey, results showed that mothers from poorer quintiles, those with low educational attainment and mothers with no gainful employment were more nutritionally at risk compared to their counterparts [5]. Thus, data suggest that the vulnerability of infants and young children and their nutritional outcomes were underscored by their mothers/caregivers' feeding practices [5]. This information is pivotal in terms of targeting the mothers and children that need more attention as well as access to the needed services and programs. In the Philippines, as reported from the 2011 to 2013 National Nutrition Surveys, the information dissemination of the importance of breastfeeding to expecting mothers has increased the rate of timely breastfeeding initiation and exclusive breastfeeding from 51.9% to 77.1%, and 48.9% to 52.3%, respectively [5]. Studies with regards to milk composition and the influence of maternal diet are generally limited but what is known poor maternal nutrition puts infants at higher risks of infections and potentially undernutrition [1].

This study evaluated the food and nutrient intakes of selected breastfeeding mothers and identified the top food sources of nutrient intakes. Researches during this period is critical to provide a basis for focused and more efficient interventions during the first thousand days of life and improve post-natal care for mothers.

## Methods

### Study Design

This is a cross-sectional, non-interventional study conducted in one of the private medical centers in Metro Manila, Philippines. The sample size included 88 mothers of healthy, term, exclusively breastfed infants aged 21–26 days at enrollment. All mothers were advised by the attending Pediatrician to have clinic visits as scheduled. Socio-economic and demographic characteristics or the general profile, like age, sex, educational level, and occupation of the mothers were collected using pre-tested questionnaires through a face-to-face interview.

#### Computation of Sample Size

The sample size was computed based on the data collected during the National Nutrition Survey 2018. The proportion of lactating mothers in National Capital Region (NCR) was 6.8%. To compute the sample we used the formula below:

$${\varvec{n}}=\frac{({\mathbf{z}}_{\propto /2}{)}^{2}\boldsymbol{*}{\varvec{p}}\boldsymbol{*}(1-{\varvec{p}})}{{\varvec{M}}{\varvec{O}}{{\varvec{E}}}^{2}}$$

where: n = total sample size.

Zα/2: 90% confidence interval, alpha 0.05 the critical value is 1.64.

p: proportion of lactating mother in National Capital Region (NCR).

MOE: margin of error (MOE) is 5.

Based on the above formula total sample size is 68. Allowing 10% drop out, the final sample size is 75.

#### Inclusion and Exclusion Criteria

The subjects in this analysis met the following criteria for inclusion: Able to speak, read, write, and understand English and Tagalog.

The mothers are physically fit with no chronic illnesses.

Is of legal age.

Mothers of healthy term, singleton infants who are between 21–26 days post-natal age at the time of enrollment in the study.

Have been breastfeeding their infants.

Willingness to comply with the study requirements.

Have read and signed the written informed consent. Mothers who cannot comply with the study protocols and did not meet the inclusion criteria were excluded from the study.

#### Recruitment Process

Potential breastfeeding mothers were referred by the attending site pediatricians, and private practitioners. Recruitment was performed at the study site during clinic visit. The informed consent (ICF) process was performed by the study physician. The mothers were also informed about the Milk Code and the advantages of breastfeeding during the discussion and collection of the informed consent.

### Dietary Assessment

Dietary assessment was collected through a face-to-face interview with the subject using a 3-day food diary.. Trained registered Nutritionist -Dietitians explained thoroughly the details in accomplishing the dietary diary using a standard instructional guidelines on how to record food intake. The quantity and types of all foods consumed over a 3-day period which included 2 weekdays and 1 weekend before the clinic visits were recorded. The mothers recorded the quantity of foods consumed using and expressed in household measures. Mothers were scheduled to visit the clinic at days 1, 22, 57, and day 90.Similarly, food diaries were obtained during these periods with a 3-day food record per clinic visit totaling 12 records per mother at the end of 90 days. An extension of the participation of the mothers until 155 days was observed for the collection and monitoring of other clinical outcomes which were recorded by Pediatricians. At every clinic visit, the records were validated face – to – face by the registered nutritionist – dietitian with the mothers.

### Anthropometric Measurement

The height and weight of the respondents were collected using a calibrated physician balance beam with a height rod (Detecto – Weigh Beam Eye-Level Scale Model 439.

400 lb x 4 oz). Measurements of both height and weight were taken twice, and the mean was considered for analysis. The body mass index (BMI) was computed by dividing the weight in kilograms by the square meter (kg / m2) height. It was interpreted using the World Health Organization (WHO) BMI classification for adults wherein the cut-off points used in classifying the nutritional status of adults 19.0 years old and above were as follows: < 18.5: Chronic Energy Deficiency/Underweight.

to 24.99—Normal

Overweight: 25.0 to 29.99.

and Obese: ≥ 30.0. (World Health Organization, 2006).

## Data processing

Quality control of the dietary intake data was conducted again by another set of registered – nutritionists – dietitians who were not involved in the data collection before encoding. Encoded data were reviewed for any inconsistencies from the source data which include the codes and quantity of consumed foods as reported including other recorded information by the mothers. Upon encoding of food records in an electronic format, it was then processed using the Individual Dietary Evaluation System (IDES) developed by the Institute to determine the estimated energy and nutrient intakes [6]. The IDES contains the updated Food Composition table which has been used in previous studies [7]. The modified Food Composition Table or FCT has 27 nutrients with 1359 food items. Approximately half of data (47%) was borrowed from the USDA National Nutrient Database and 39% data from the initial Filipino FCT archived by DOST—FNRI. The rest data were borrowed from the food composition database of ASEAN and other Asian countries such as Japan (8%) and information from food labels (6%). All borrowed data were adapted according to the FAO INFOODS guidelines (International Network of Food Data Systems (INFOODS) 2012 [8].

Prior to data processing, encoded data were checked to identify implausible values by adopting the method described by Lopez-Olmedo and colleagues [9]. The estimated energy requirement (EER) was calculated for each individual by using the equations for maintenance of body weight from the Institute of Medicine based on age, gender, weight, height, and physical activity information [10]. Since physical activity was not measured in the study, we assumed a sedentary physical activity level for this study population. The ratio of daily energy intake to EER for each person was calculated for implausible energy intake and converted to a logarithmic scale to eliminate outliers under -3 SDs and over + 3 SDs. Excessive intakes indicated if it exceeds 1.5 times the 99th percentile of nutrient intake for improbable micronutrient intake distribution observed in the equivalent variable sex and age group. Intakes higher than upper limit were replaced by a random value produced at intervals with a lower bound equal to the 95th percentile of the observed intake and an upper bound equal to 1.5 times the 99th percentile of the uniform distribution. [9].

## Statistical Analysis

Descriptive analysis was used to compute the mean, standard deviation (SD), percentages, and proportions to summarize socio-demographic variables.

The mean usual energy and nutrient intake distributions of the 12 food records for each subject were calculated using software established by Iowa State University for each subject, PC-SIDE version 1.02 (Iowa State University, Ames, IA, USA) [11], for which an in-person difference in nutritional intake over days was accounted for. The percentiles of the usual nutrient intake distributions are estimated by this software, as well as the proportion below the estimated average requirements (EAR) identified by the Philippine Dietary Reference Intakes 2015. Inadequacy is calculated as the proportion of people with usual intakes below the EAR [6].

Acceptable Macronutrient Distribution Ranges (AMDR) were used to evaluate carbohydrates, total fat, and protein intakes as a percentage of energy. The proportion of insufficient and excessive intakes was classified as lower values and greater than AMDR upper values. A probability approach method was used for the determining iron intake deficiency [12]. First, the risk of inadequacy of each person was determined, followed by the prevalence of insufficient iron intake, which is the average risk of inadequacy. Calculations for summary statistics were carried out using STATA version 13 [13].

Food group intake was calculated by the percentage of mothers who at least once consumed particular foods or food groups on the first 24-h dietary record, regardless of the amount consumed. This method has been used in our previous studies [14].

Determination of the weighed percentage contribution of each food group relative to the selected key nutrient was done by the addition of the amount of nutrient present in all individuals by each food group divided by the cumulative intake of that nutrient consumed by all subjects from all varieties of food and beverages.

## Results

The study started with a sample size of eighty-eight mothers, five [1] dropped out from the study because they did not continue after the first clinic visit while 13 had incomplete data because of irregular clinic visits, thus only records of the 70 respondents were included in the analysis which is still adequate to meet the computed sample size of 68. In Table 1 the mean age of breastfeeding mothers was 26.9 years (SE = 0.7) with a mean weight of 56.9 kg (SE = 1.3) and a mean height of 152.4 cm (SE = 0.6). In terms of body mass index (BMI), 8.6% were chronic energy deficient, and 34.3% are overweight while 12.9% were obese. The occupation of the breastfeeding mothers were mostly housewives (41%) and 28% were working under the category of service/shop/market sales. Educational attainment of most mothers indicates a high school level of education (42%). There was a similar percentage of male and female infants and the mean birth weight for male and female infants are 3112.0 g and 3111.0 g respectively.

**Table 1:** Demographic characteristics of breastfeeding mothers (*n* = 70)

Variables	Mean ± SE
	***n* = 70**
**Age, (years)**	26.9 ± 0.7
**Weight, (kg)**	56.9 ± 1.3
**Height, (cm)**	152.4 ± 0.6
**BMI, *n (%)***	
CED	6 (8.57)
Normal	31 (44.3)
Overweight/Obese	33 (47.1)
**Occupation, *n (%)***	
Clerk	1 (1.4)
Housewife	41 (58.6)
Service/shop/market sales	28 (40.0)
**Education Attainment, *n (%)***	
Elementary level	2 (2.9)
High school level	42 (60.0)
College level	8 (11.4)
Associate degree (2 years college)	12 (17.1)
Vocational level	6 (8.6)
**Infant Gender, *n (%)***	
Male	38 (54.3)
Female	32 (45.7)
**Infant Birth weight, (g)**	3092.6 ± 39.1

The usual nutrient intake of breastfeeding mothers is shown in Table 2. The mean energy intake was 2516.7 ± 63.2 kcal/day. The mean for macronutrients is as follows: carbohydrates (404 g/d).

**Table 2:** Usual nutrient intake of breastfeeding mothers (*n* = 70)

	**Dietary Reference Intakes**^**b**^		**Mean/Median Intake Percentiles**						**Inadequate/Excessive Reported Intake**	
**Nutrients**	**EAR/AMDR**	**UL**	**10th**	**25th**	**Median**	**Mean ± SE**	**75th**	**90th**	**% < EAR/** **AMDR**	**% > AMDR** **/ > UL**
**Macronutrients**										
Energy intake (kcal)	1957 (EER)^**a**^	-	1903	2152	2459	2516.7 ± 63.2	2815	3200	13	-
Total fat (g/d)	-	-	41	50	62	64.5 ± 2.4	76	91	-	-
Saturated fat (g)	-	-	17	19.9	23.8	25.2 ± 0.9	28.9	34.9	-	-
Monounsaturated fatty acids (g)	-	-	13.3	16.7	21.3	22.5 ± 0.9	27	33.3		
Polyunsaturated fatty acids (g)	-	-	6.7	8.1	10	10.5 ± 0.4	12.4	15	-	-
Protein (g/d)	72	-	59.9	67.5	77	78.4 ± 1.8	87.8	98.6	37	-
Carbohydrate (g/d)	-	-	302	344	397	404.2 ± 10.2	456	517	-	-
Total sugars (g/d)	-	-	37	47	60	63.7 ± 2.9	76	95	-	-
Dietary fibre (g/d)	-	-	8.1	9.3	10.8	11.1 ± 0.3	12.6	14.4	-	-
**As percentage of total energy**										
Total Fat (%)	15–30	-	16.9	19.2	21.9	22.2 ± 0.5	24.9	27.8	3	4
Protein (%)	10–15	-	11.7	12.1	12.7	12.7 ± 0.1	13.2	13.7	0	0
Carbohydrate (%)	55–75	-	59	62.1	65.4	65.1 ± 0.6	68.4	70.9	2	1
**Micronutrients**										
Vitamin C (mg/d)	-	-	18	26	37	43.5 ± 3.1	54	76	-	-
Vitamin E (mg)	-	-	2.5	3.2	4.1	4.4 ± 0.2	5.3	6.7	-	-
**B vitamins**										
Thiamine (mg/d)	1.1	-	1	1.1	1.4	1.4 ± 0.1	1.7	2	22	-
Riboflavin (mg/d)	1.3	-	0.9	1.1	1.4	1.6 ± 0.1	1.8	2.4	39	-
Niacin (mg/d)	13.4	35	18.1	20.7	24.3	26.0 ± 0.9	29.3	35.8	0	11
Vitamin B6 (mg)	1.7	100	1.2	1.4	1.6	1.6 ± 0.04	1.8	2.1	63	0
Folate (DFE μg)	405	1000	194	231	280	290.9 ± 9.9	339	401	96	0
Vitamin B12 (mg)	2.4	-	1.7	2	2.5	2.7 ± 0.1	3.1	3.8	46	-
**Bone-related nutrients**										
Calcium (mg/d)	-	-	343	441	585	644.7 ± 34.8	781	1018	**-**	-
Phosphorus (mg/d)	580	4000	883	1016	1182	1220.5 ± 35.4	1382	1604	< 1	0
Magnesium (mg)	-	-	157	182	214	222.2 ± 6.8	254	297	**-**	-
Vitamin D (mg)	-	-	1.9	2.5	3.3	3.5 ± 0.2	4.3	5.4	**-**	-
**Other micronutrients**										
Vitamin A (μg RE/d)	-	-	364	466	614	695.2 ± 42.4	827	1111	**-**	-
Iron (mg/d)	28.2	45	8.8	9.9	11.4	11.9 ± 0.9	13.3	15.5	99	0
Zinc (mg)	-	-	6.3	7.2	8.3	8.5 ± 0.2	9.7	11.1	**-**	-
Sodium (mg/d)	-	-	1135	1349	1628	1684.7 ± 56.1	1958	2306	**-**	-
Potassium (mg)			1150	1309	1513	1550.2 ± 40.5	1751	1999	-	-
Selenium (mg)	35.3	400	98	108	121	122.6 ± 2.4	135	149	-	-

^b^*PDRI* Philippine Dietary Reference Intakes 2015, *EAR* estimated average requirements, *AMDR* acceptable macro-nutriment distribution range, *UL* tolerable upper intake level

^a^energy intake cutoffs based on Estimated Energy Requirement (IOM methods) assume their physical activity was low active

protein (78.4 g/d) and total fat (64.5 g/d). In terms of the macronutrient intake percent distribution, 37% of the mothers had protein intake below the EAR/AMDR.

3% had a percentage of energy from total fat below the EAR/AMDR while 4% of the mothers exceeded the EAR/AMDR. Furthermore, with respect to the percentage of energy from carbohydrates, 2% of the breastfeeding mothers have intakes below EAR/AMDR while 1% exceeded the EAR/AMDR. High prevalence of inadequate micronutrient intakes was also observed: iron (99%), folate (96%), and Vitamin B6 (63%), Vitamin B12 (46%), riboflavin (39%) and thiamine (22%). Moreover, about 11% of mothers had excessive niacin intake or above the EAR/AMDR. Mean selenium intake (122.6 mg) was above the EAR/AMDR (35.3 mg).

**Fig. 1:**
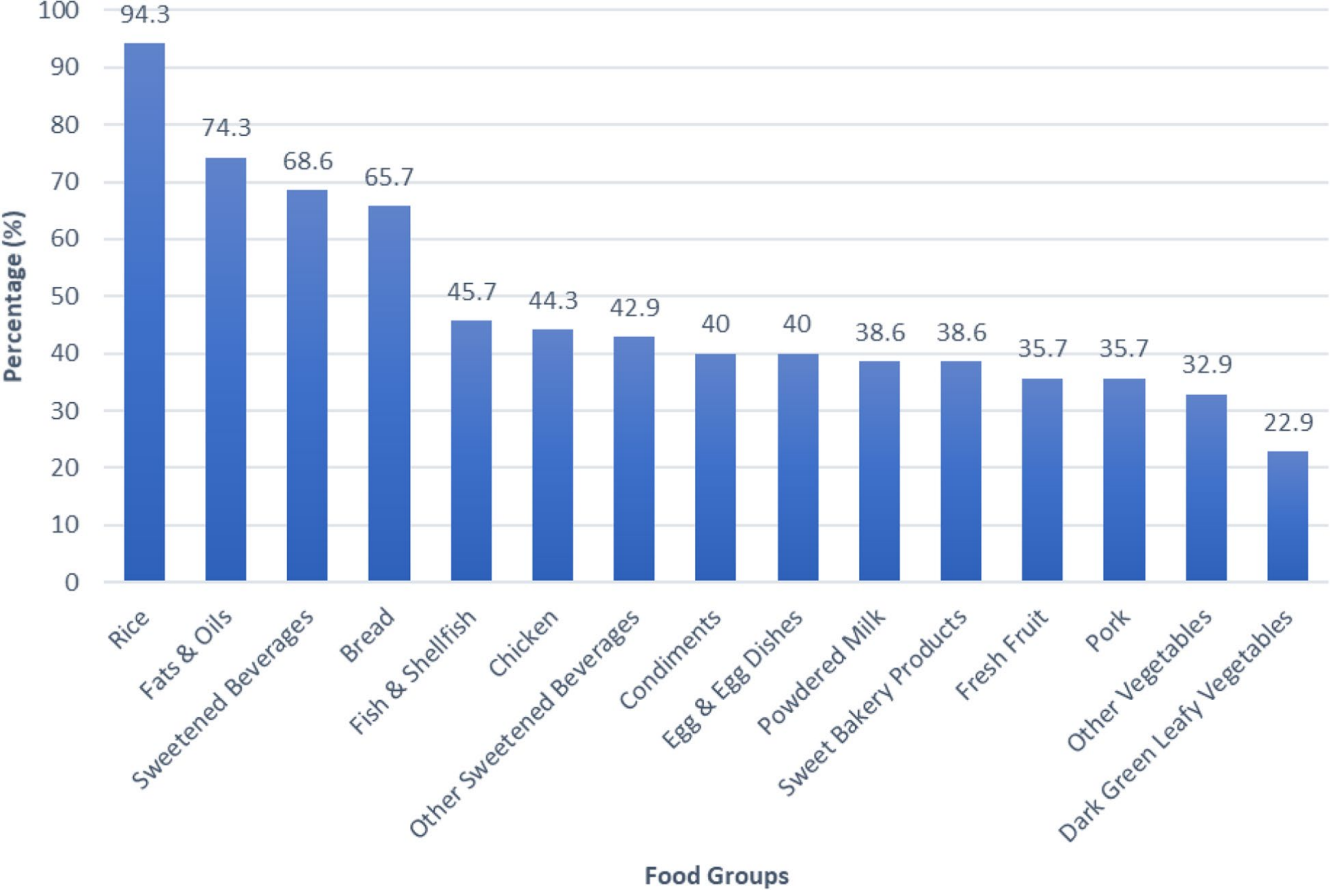
Top 15 most consumed foods of breastfeeding mothers

Rice (94.3%), fats and oils (74.3%), sweetened beverages (68.6%), bread (65.7%), and fish & shellfish (45.7%) were the top-5 most consumed foods of breastfeeding mothers as seen in Fig. 1. Dark green leafy vegetables were the least consumed food group of breastfeeding mothers (22.9%).

**Fig. 2:**
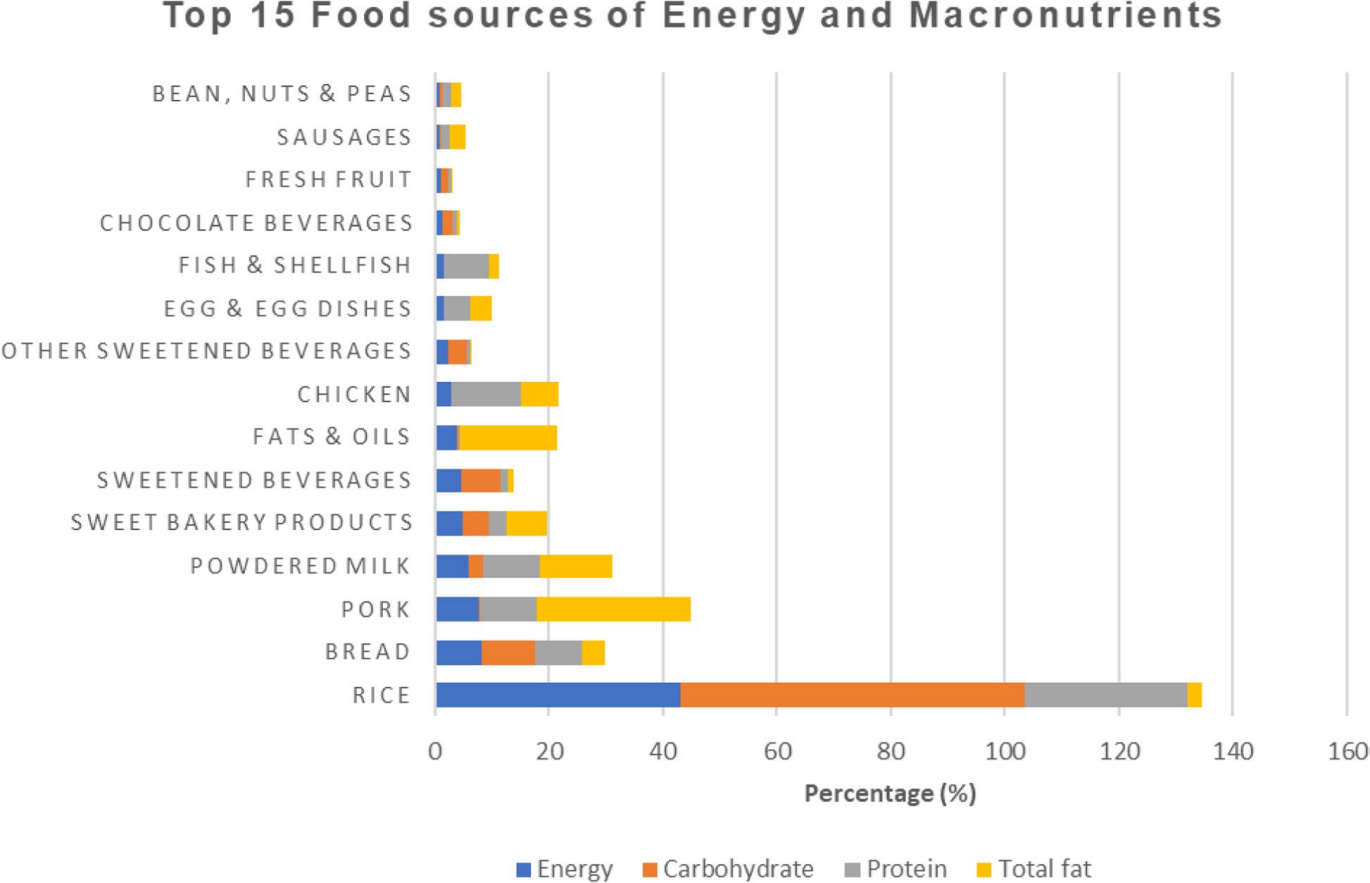
Contribution of top 15 most consumed food to sources of Energy, Carbohydrate, Protein and Total fat (*n* = 70)

As seen in Fig. 2, rice, sweetened beverages, bread, powdered milk, and pork were the top-5 sources of macronutrients such as carbohydrates, protein, and fats. Briefly, rice is the major source of carbohydrate (60.4%), protein (28.6%), thiamine (21.8%), iron (26.4%), and zinc (28.5%), a second source of calcium and third source of riboflavin. Pork was the first source of total fat (27.1%), a second source of zinc (13.7%), and the third source of thiamine (12.7%). Sweetened beverages were the first source of vitamin C (35.5%), the second source of thiamine (16.7%), zinc (14.0%), riboflavin (12.5%), and third source of vitamin A (10.9%) as well as carbohydrate (6.8%). Other food groups were consumed by percentages ranging from 15.7–38.6% and contributed too little in the overall nutrient intakes.

**Fig. 3:**
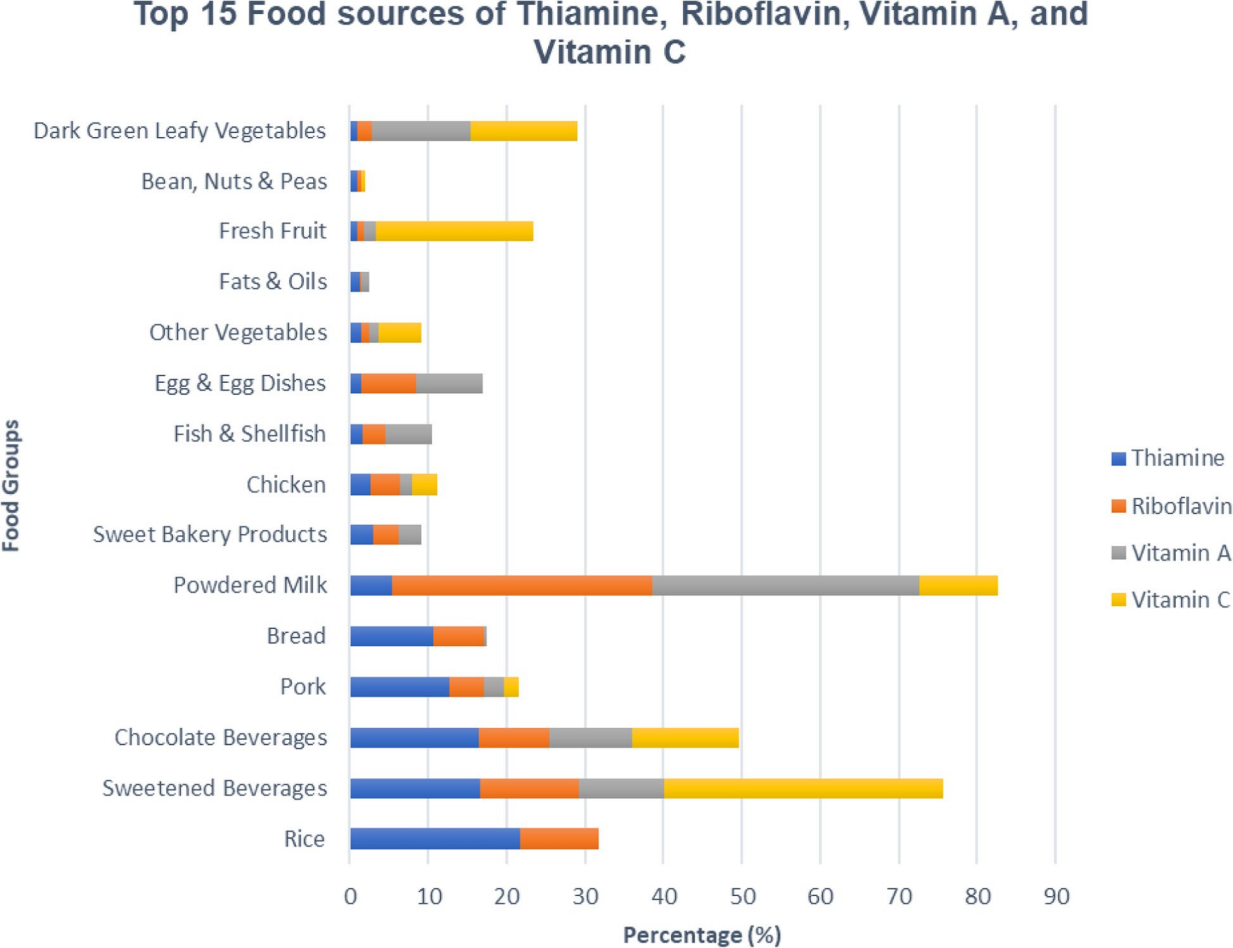
Contribution of Top 15 food sources of Thiamine, Riboflavin, Vitamin A, and Vitamin C

Top 5 food sources of thiamine from foods consumed by breastfeeding mothers are from rice (21.8%), Sweetened Beverages (16.7%), Chocolate Beverages (16.5%), Pork (12.7%) and Bread (10.7%) as seen in Fig. 3. It can also be noted that top food sources for Vitamin C come mainly from sweetened beverages (35.5%) and Fresh fruit. On the other hand, top food sources for riboflavin and vitamin A are from powdered milk with 33.2% and 34.1% consumption respectively.

**Fig. 4:**
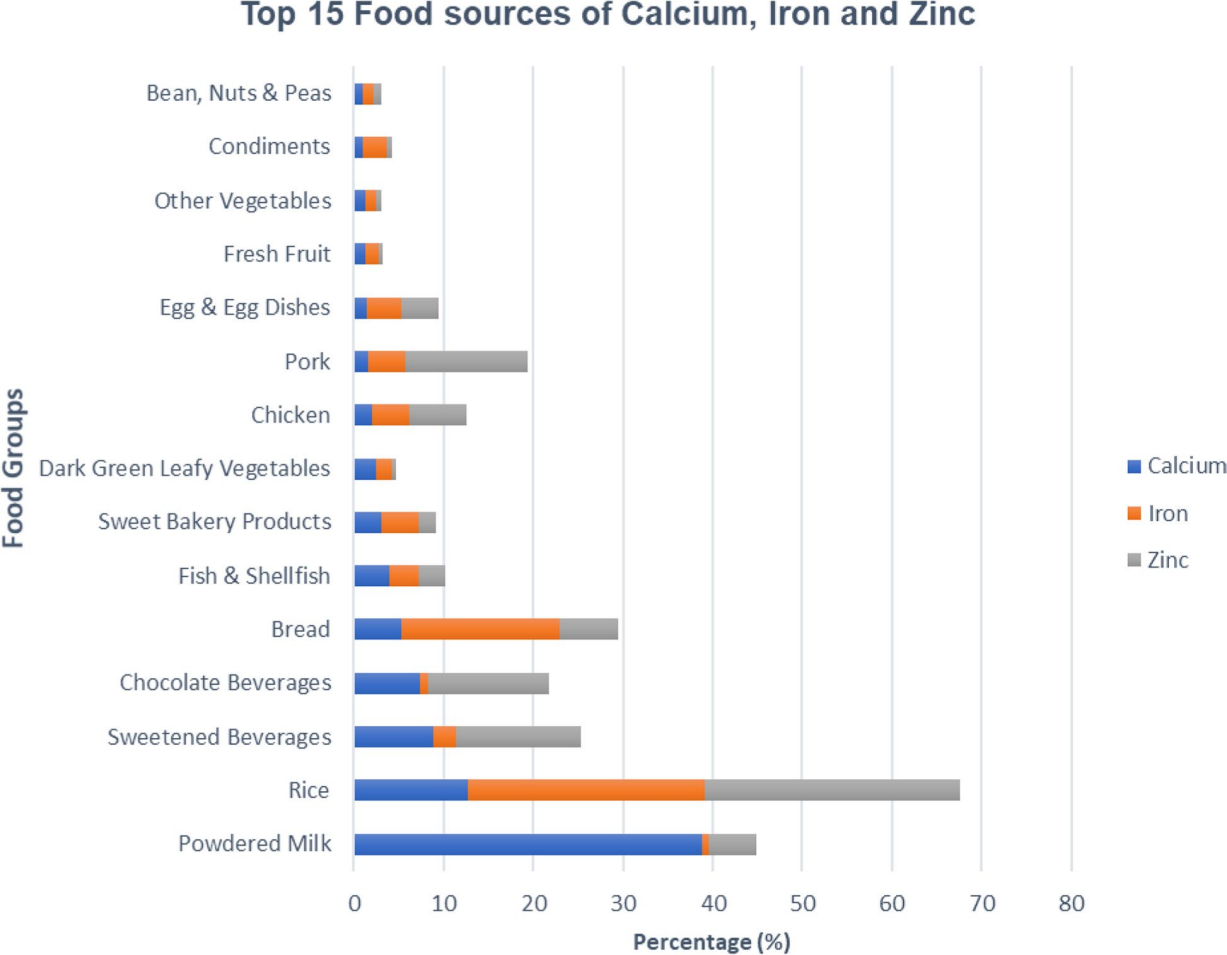
Contribution of Top 15 food Sources of Calcium, Iron and Zinc

As seen in Fig. 4, top food sources of calcium from breastfeeding mothers come mainly from powdered milk (38.8%), rice (12.7%), and sweetened beverages (8.9%). While food sources of iron come mainly from rice (26.4%) and bread (17.7%), food sources for zinc mainly come from rice (28.5%), sweetened beverages (14.0%), and pork (13.7%).

## Discussion

The study focused on maternal food sources and nutrient intakes of selected breastfeeding mothers. The marked inadequacies of protein, and micronutrients puts breastfeeding mothers in a critical state of malnutrition which may have an impact on the young breastfed infant. Many studies have shown the association of maternal diet and the quality of breastmilk. In one study it has been revealed that maternal dietary patterns can affect macronutrient intake as well as breastmilk's fatty acid profiles [15]. Moreover, breastmilk content of some vitamins and minerals such as thiamine, riboflavin, vitamin B6, and vitamin B12 were found to be low in mothers with these nutritional deficiencies [16].

Inadequate protein intake was reported among 37% of the breastfeeding mothers in this study. Protein inadequacy among breastfeeding mothers is very critical because it has been founded in previous studies that a low protein diet reduces prolactin secretion which can affect milk production [17]. In a similar study [18], poor maternal nutrition especially protein restriction during lactation directly affects the weight of infants wherein it was found out that breastfeeding mothers with restricted protein in their diets during lactation had infants who weighed less compared to its counterpart. This significantly emphasizes the role of protein in the maternal diet in the early stages of life [17, 18].

In addition, results showed a high incidence of insufficient intake of micronutrients, especially for iron (99%), Folate (96%), and Vitamin B6 (63%). Based on previous studies, current dietary iron intakes of breastfeeding mothers are unable to meet the requirements, and even iron fortification is not sufficient to fill the needs of breastfeeding mothers [19, 20]. Iron deficiency may, in turn, cause long term cognitive and motor impairments to the infant that heavily relies on maternal stores since iron plays a critical role in cellular function, brain growth and development through its supporting role in neuronal and glial energy metabolism [16]. On the other hand, deficiency in vitamin B6 can affect breastfeeding since maternal intake is relative to breast milk concentration [21, 22]. This is long-established by the experts' consultation report of the World Health Organization wherein it states that maternal deficiency in vitamin A and B6 can ultimately affect the nutrient status of infants who mainly rely on breastmilk as their source of nutrition [23]. Vitamin B6 deficiency in infants may cause problems in a nucleic acid synthesis which is directly related to the formulation of brain structure.

thus, brain development may be impaired if deficiency arise [23]. In terms of folate intake, maternal folate stores are mobilized into breast milk to sustain their secretion levels, thus breastfeeding women with low intakes will have severely low levels of nutrients as breastfeeding progresses [16]. Folate deficiency may progress into megaloblastic anemia and is critical also for the next pregnancy because maternal folate deficiency may lead to neural tube defects for the developing fetus [24, 25]. Moreover, a considerable high prevalence of inadequate micronutrient intakes was found for Vitamin B12 (46%), Riboflavin (39%), and Thiamine (22%). These nutrients also have a direct correlation between maternal intakes and infant nutrient status as stated in several studies, wherein infant vitamin B12 status is affected by breast milk concentration of vitamin B12 in breastfed infants aged 0 to 6 months [22, 26, 27]. For thiamine, breast milk concentrations, as well as infant status, are heavily dependent on the mother's intake and nutritional status [28]. Furthermore, a previous report shows that severe thiamine deficiency leads to reduced infant growth [29]. The results of this study are aligned with previous studies and surveys, in which suboptimal energy and nutrient intakes are reported in breastfeeding mothers emphasizing the vulnerability of breastfeeding mothers to energy and nutrient inadequacies despite the increased nutrient requirements during this stage of life [30–33].

It is therefore of prime importance to install programs uplifting the health and nutritional status of breastfeeding women as they are the source of a better quality of breastmilk. From infancy to early childhood years, breastmilk poses an important source of energy and various nutrients through children 6–23 months of age as it can provide most of the child's energy needs, and one-third of the required energy needs for children between 12 and 24 months [34].

In our study the mean energy intake (2516.7 ± 63.2 kcal/day) of mothers which was 28.6% higher than the estimated EER (1957 kcal/day) might increase the possibility of being overweight/obese. National Nutrition Survey results from 2011 to 2015 have highlighted that the prevalence of Chronic Energy Deficiency and underweight among lactating mothers slightly increased from 11.9% to 12.5%, while overweight barely changed from 17.7% prevalence to 17.5%. This highlights a double burden of malnutrition among breastfeeding mothers encompassing the lack of energy and micronutrients of their diets. According to Fikawati et al. (2014), sufficient nutrient and caloric intake are needed to suffice milk production, recovery after childbirth, maintenance of breastmilk quantity, and quality in its premise to combat maternal malnutrition [35]. However, knowledge dissemination or nutrition education must be intensified so as not to overdo the intake of high caloric foods and improving the variety and nutrient density of the diet, while limiting excessive caloric intake are key interventions. It should be noted that excessive fat intake was also observed in this group of mothers. Uncorrected food patterns may result in cardio-metabolic disorders like diabetes, hypertension, and other vascular diseases. The 2018 National Survey results showed that prevalence of diabetes was 4.2% and hypertension was 18.0% among adults 20 years and over were among the recorded pregnancy complications experienced by this age-group. Moreover, elevated Blood Pressure and impaired and elevated fasting blood sugar affected 2.97% of pregnant women and 5.32% of breastfeeding mothers as reported in the 2013 NNS. The results from the past surveys emphasize the high risk for heart disease and diabetes among pregnant and lactating women, and therefore dietary interventions that can provide solutions to these problems should be considered. While recommendations are increased to meet the demands of lactation, it is equally important not to exceed the recommendations beyond their values in order to prevent accumulation of excessive fat and cause excessive weight gain that is difficult to reverse beyond the period of exclusive breastfeeding.

In terms of commonly consumed foods, rice is the first source of energy and the top source of many key nutrients. The results are consistent with the survey results from the 2018 Expanded National Nutrition Survey wherein it was found out that the top food source for total energy intake among lactating mothers comes from rice (58.1%) [36]. This may be due to rice being a staple in the Philippines. However, besides fish and vegetables, the mothers’ diet lacks other nutrient-dense foods. The study found that overall, fruits (35.37%) and vegetables (18.6–32.9%) were one of the least consumed food groups by breastfeeding mothers (*n* = 70) and contributed little nutrient intake. This is in conjunction with the study of Shah et al. (2010) which showed low fruit and vegetable consumption, an area of concern identified by HEI-2005, specifically among low-income, early postpartum women [37]. In comparison to existing dietary food guidelines in the Philippines such as the *Pinggang Pinoy,* which is a Filipino food plate model to convey the right food group proportions per meal basis to meet energy and nutritional demands among lactating women, the results from the study are far from the idle recommendations [38]. In a per meal basis, the recommendation to consume cooked vegetables is around 1- ½ cups for lactating women. However, the results stipulated that there is low consumption of vegetables. Mean fiber intake was only 11 g/day, which is lower than the recommended intake (RNI: 20–25 g) which is detrimental to digestive and cardiovascular health among breastfeeding mothers. Furthermore, fruits and vegetables not only provide good sources of fiber for digestive health but they are also low in energy density but nutritionally dense with vitamins and minerals such as Vitamin C, D, Calcium, and Potassium [39]. In addition, other nutrient-rich foods such as egg and egg dishes (40.0%) and pork (35.7%) were not that commonly consumed either. Poor consumption of egg/egg dishes and pork which are iron-rich foods can be a factor for the high prevalence of iron deficiency (99%) among breastfeeding mothers (*n* = 70) [1]. Protein recommendations for breastfeeding mothers in the *Pinggang Pinoy* equates to about 1–2 pieces of medium variety of dish or 2 pieces of medium chicken leg and equivalents per meal ideally to support protein and nutrient needs in this life stage [38].

Sugar-sweetened beverages (SSB) were among the top sources of nutrients such as vitamin C, calcium, iron, zinc, thiamine, riboflavin, and vitamin A in the diets of breastfeeding mothers. Sugar-sweetened beverages are affordable in the Philippines and are one of the most commonly consumed foods. Thus, these are often used as shuttles for fortification in foods [40–42]. This makes sugar-sweetened beverages a source of micronutrients in this population, although overconsumption may increase the risk of obesity as these are rich in high fructose corn syrup that disables insulin secretion and do not enhance leptin production contributing to high energy intake and weight gain [43]. To date, the consumption of SSB is controlled because of the Sin Tax Law.

Strengths: For each participant in the study, 12 food records were collected and analyzed providing a very detailed and strong foundation to assess the nutrient intake. These were collected by trained interviewers, and validated with the subject face to face ensuring no loss of information. In addition, strict quality control was employed to deal with implausible data and removing all outliers. This study is only one of the very few studies reporting the detailed food and nutrient intake very extensively among lactating Filipino women.

Limitations: Added sugar was not evaluated in this study since the data currently not available in the Philippine Food Composition Table (FCT) database. This study also recognizes that the data available only features the intakes of breastfeeding mothers until 90 days which is less than the recommended period of 6 months for exclusive breastfeeding. Furthermore, the nutrient status and human milk compositions were not reported in the study which would have allowed a better understanding of the impact of inadequacies on maternal nutrient deficiencies based on both blood and milk biomarkers. Future research could also take into account other factors that could affect maternal intakes such as socioeconomic factors, financial capability, food consumption habits, and food availability.

## Conclusion

This study provides adequate information that nutrient inadequacy is common among breastfeeding mothers which might be attributed to the consumption of poor quality of food sources of nutrients. The consumption of high calorie dense foods might be the result of the high prevalence of overweight and obesity. This study can serve as a scientific reference for developing policies and interventions aimed at improving the food intake during breastfeeding which is beneficial for both mother and child.

## Supplementary Information

**Additional file 1:Table S1.** Contribution of top 20 consumed food to energy and selected nutrient intakes of breastfeeding mother

## Data Availability

Additional supporting material may be available upon request from the Authors.

## Abbreviations

EAR: Estimated Adequate Requirement
AMDR: Acceptable Macronutrient Distribution Ranges
EER: Estimated Energy Requirements
WHO: World Health Organization
FCT: Food Composition Table
BPO: Business Process Outsourcing
BMI: Body Mass Index
IOM: Institute of Medicine
AI: Adequate Intake
RNI: Recommended Nutrient Intakes
FNRI: Food and Nutrition Research Institute
CIOMS: Council for International Organizations of Medical Sciences
FIERC: FNRI Institutional Ethics Review Committee
ICF: Informed Consent Form
CED: Chronic Energy Deficiency
HEI: Healthy Eating Index
SSB: Sugar-sweetened Beverages
