# Supplementary material for: Analysis of food sources and nutrient intakes of selected breastfeeding mothers in Metro Manila, Philippines
Source: BMC Nutr. 2022 Jan 18;8:6. doi: 10.1186/s40795-022-00502-1 (PMC8764776; doi:10.1186/s40795-022-00502-1)
Supplement: Supplementary file 1 — Additional file 1:Table S1. Contribution of top 20 consumed food to energy and selected nutrient intakes of breastfeeding mother [file 40795_2022_502_MOESM1_ESM.docx]

**Table S1**. Contribution of top 20 consumed food to energy and selected nutrient intakes of breastfeeding mother

|  |  |  |  | **Percent contribution to total daily intake** | | | | | | | | | | | |
| --- | --- | --- | --- | --- | --- | --- | --- | --- | --- | --- | --- | --- | --- | --- | --- |
|  |  |  |  | **Macronutrients** | | | | | **Vitamins** | | | **Minerals** | | | |
| **Rank** | **Food groups** | **% Consuming (n=70)** | **Mean intake per capita [g]** | **Energy** | **Carbohydrate** | **Protein** | | **Total fat** | **Thiamine** | **Riboflavin** | **Vitamin A** | **Vitamin C** | **Calcium** | **Iron** | **Zinc** |
| 1 | **Rice** | 94.3 | 321.3 (5.8) | 43.1 | 60.4 | 28.6 | 2.5 | | 21.8 | 9.9 | 0 | 0 | 12.7 | 26.4 | 28.5 |
| 2 | **Fats & Oils** | 74.3 | 15.1 (1.7) | 4 | 0.1 | 0.3 | 17 | | 1.3 | 0.2 | 1 | 0 | 0.4 | 0.5 | 0.4 |
| 3 | **Sweetened Beverages** | 68.6 | 66.2 (14.6) | 4.7 | 6.8 | 1.3 | 0.9 | | 16.7 | 12.5 | 10.9 | 35.5 | 8.9 | 2.4 | 14 |
| 4 | **Bread** | 65.7 | 66.0 (3.2) | 8.1 | 9.6 | 8.1 | 4.1 | | 10.7 | 6.4 | 0.3 | 0 | 5.3 | 17.7 | 6.4 |
| 5 | **Fish & Shellfish** | 45.7 | 34.9 (4.9) | 1.5 | 0.1 | 7.9 | 1.9 | | 1.6 | 3 | 5.9 | 0 | 3.9 | 3.3 | 2.9 |
| 6 | **Chicken** | 44.3 | 50.5 (4.8) | 3 | 0 | 12 | 6.7 | | 2.7 | 3.7 | 1.6 | 3.1 | 2 | 4.2 | 6.3 |
| 7 | **Other Sweetened Beverages** | 42.9 | 18.7 (4.1) | 2.3 | 3.3 | 0.7 | 0.2 | | 0 | 2.3 | 0 | 0 | 0.5 | 0 | 0.3 |
| 8 | **Condiments, Sauces, Herbs, Spices and Other Seasoning** | 40 | 15.7 (2.6) | 0.6 | 0.9 | 0.6 | 0 | | 0.2 | 0.4 | 0.3 | 1 | 1 | 2.7 | 0.6 |
| 9 | **Egg & Egg Dishes** | 40 | 30.0 (4.0) | 1.5 | 0.1 | 4.5 | 4 | | 1.4 | 7.1 | 8.4 | 0 | 1.4 | 3.9 | 4.1 |
| 10 | **Milk Powdered** | 38.6 | 30.8 (5.1) | 5.9 | 2.7 | 9.8 | 12.7 | | 5.3 | 33.2 | 34.1 | 10.1 | 38.8 | 0.7 | 5.4 |
| 11 | **Sweet Bakery Products** | 38.6 | 32.7 (4.1) | 5 | 4.6 | 2.9 | 7.1 | | 3 | 3.2 | 3 | 0 | 3.1 | 4.1 | 2 |
| 12 | **Fresh Fruit** | 35.7 | 36.0 (5.8) | 1 | 1.4 | 0.4 | 0.1 | | 1 | 0.8 | 1.5 | 20.2 | 1.3 | 1.5 | 0.4 |
| 13 | **Pork** | 35.7 | 55.0 (5.1) | 7.7 | 0.3 | 9.8 | 27.1 | | 12.7 | 4.4 | 2.5 | 1.9 | 1.5 | 4.2 | 13.7 |
| 14 | **Other Vegetables** | 32.9 | 31.3 (3.6) | 0.4 | 0.5 | 0.6 | 0.1 | | 1.4 | 1.1 | 1.1 | 5.5 | 1.2 | 1.3 | 0.6 |
| 15 | **Dark Green Leafy Vegetables** | 22.9 | 15.4 (2.5) | 0.3 | 0.2 | 0.7 | 0.1 | | 1 | 1.9 | 12.5 | 13.6 | 2.5 | 1.8 | 0.4 |
| 16 | **Sausages** | 21.4 | 10.6 (3.5) | 0.9 | 0.1 | 1.7 | 2.7 | | 0.3 | 0.7 | 0.1 | 0 | 0.2 | 5.1 | 1.8 |
| 17 | **Starchy Vegetables** | 18.6 | 8.1 (1.6) | 0.2 | 0.3 | 0.2 | 0 | | 0.6 | 0.3 | 0 | 5 | 0.4 | 0.6 | 0.2 |
| 18 | **Sugar** | 17.1 | 2.1 (0.8) | 0.3 | 0.5 | 0 | 0 | | 0 | 0 | 0 | 0 | 0.1 | 0 | 0 |
| 19 | **Bean, Nuts & Peas** | 17.1 | 5.5 (2.2) | 0.9 | 0.5 | 1.5 | 1.7 | | 1 | 0.4 | 0.1 | 0.5 | 1 | 1.1 | 1 |
| 20 | **Chocolate Beverages** | 15.7 | 9.2 (5.4) | 1.4 | 1.8 | 0.6 | 0.6 | | 16.5 | 9 | 10.5 | 13.7 | 7.3 | 1 | 13.4 |
| **Total** | | | | 92.8 | 94.2 | 92.2 | 89.5 | | 99.2 | 100 | 93.8 | 110.1 | 93.5 | 82.5 | 102.4 |
